# MOF Regulates TNK2 Transcription Expression to Promote Cell Proliferation in Thyroid Cancer

**DOI:** 10.3389/fphar.2020.607605

**Published:** 2020-12-08

**Authors:** Danyang Li, Yang Yang, Bo Chen, Xinghong Guo, Shuang Gao, Meng Wang, Mingxiao Duan, Xiangzhi Li

**Affiliations:** ^1^ Shandong Provincial Key Laboratory of Animal Cell and Developmental Biology, School of Life Sciences, Shandong University, Qingdao, China; ^2^ Rehabilitation Center, Qilu Hospital, Cheelo College of Medicine, Shandong University, Jinan, China; ^3^ School of Pharmacy, Binzhou Medical University, Yantai, China; ^4^ Department of Thyroid Surgery, Qilu Hospital, Cheelo College of Medicine, Shandong University, Jinan, China; ^5^ Department of Endocrinology, Qilu Hospital, Cheelo College of Medicine, Shandong University, Jinan, China

**Keywords:** thyroid cancer, MOF, TNK2, transcription, proliferation

## Abstract

MOF is a well-known histone acetyltransferase to catalyze acetylation of histone H4 lysine 16 (K16), and it is relevant to diverse biological processes, such as gene transcription, cell cycle, early embryonic development and tumorigenesis. Here, we identify MOF as an oncogene in most thyroid cancer. It is found that expression level of MOF was significantly upregulated in most thyroid cancer tissue samples and cell lines. MOF-deficient in both BHP-10-3 and TT2609 cell lines inhibited cell proliferation by blocking the cell cycle in G1 phase and enhanced cell apoptosis. Mechanistically, MOF bound the TNK2 promoter to activate TNK2 transcription. Furthermore, the expression level of TNK2 was decreased with the histone acetyltransferase inhibitor. Besides, MOF promoted proliferation of thyroid cancer cells through increased phosphorylation of AKT, thus activating the PI3K/AKT pathway. Ultimately, our findings indicated that MOF played an oncogene role in development and progression of thyroid cancer and may be a potential novel target for the treatment of thyroid cancer.

## Introduction

Thyroid cancer is the most common malignant tumor of the head and neck with high morbidity. According to the statistics of the National Cancer Center in 2018, thyroid cancer has become the fourth most malignant tumor in women. Although most patients have a favored prognosis after surgery and radioactive I^131^ treatment, treatment failure and inevitable side effects still exist. Therefore, molecular targeted therapy is of great significance. Current research confirms that some genetic mutations occur in up to 97% thyroid cancer (Cancer Genome Atlas Research Network, 2014). Most thyroid cancers are closely related to MAPK, VEGF, and PI3K signaling pathways, while BRAF, RAS and RET genes have higher mutation rates, which provides molecular targets for thyroid cancer (Thomas et al., 2014). Six molecular targets of thyroid cancer including BRAF, RET, NTRK1, G-GAS, K-RAS, and N-RAS have already been reported (Xing, 2013). Though BRAF inhibitor sorafenib has been used clinically, others are still in the basic research or clinical trial phase (Thomas et al., 2014).

Epigenetics is a hot topic in recent years, and epigenetic modifications have been observed in most tumors (Verma et al., 2014). Epigenetic modifications are also common with thyroid cancer. In follicular thyroid cancer, the promoter of PTEN is usually methylated, and the degree of methylation is inversely related to the degree of tumor differentiation (Hou et al., 2008). At present, there is no clear explanation for the modification of histone acetylation for thyroid cancer. However, treatment of thyroid cancer cells with HDAC inhibitors alters the expression levels of some genes involved in thyroid cancer differentiation (Sherman et al., 2013). There is still a large gap in the study of non-coding RNA in thyroid cancer compared to other epigenetic modifications. The current study confirmed that PTCSC3 is a thyroid-specific LncRNA whose expression level is significantly down-regulated in papillary thyroid cancer (Fan et al., 2013). Changes in epigenetic modifications are detectable in the early stages of development, progression, eventual recurrence and metastasis. It has a great significance to clarify and use these markers to identify groups of at-risk patients, improve diagnostic criteria, and guide treatment options and predictors (Pan et al., 2018). In addition, the targeted locus of epigenetic modification provides new ideas for changing treatment regimens and provides new treatment options for patients with abnormal epigenetic modifications.

The overall level of acetylation of cells is maintained by histone acetyltransferases (HATs) and histone deacetylases (HDACs), which is in dynamic equilibrium and plays an important role in the regulation of chromatin structure and function (Kouzarides, 2007). HATs are divided into two families. One is GCN5-mediated N-acetyltransferase (GNATs), including GCN5 and PCAF (Dyda et al., 2000). Another is MYST family, including MOF, TIP60, HBO and others.

MOF, also known as MYST1 or KAT8, is a member of MYST family. The highly conserved MYST domain determines its histone acetyltransferase activity. MOF has a strict substrate specificity for the histone H4 at lysine 16 (H4K16), which was first discovered and studied in *drosophila*. As an important component of the *drosophila* X chromosome dose-compensating complex called male special lethal (MSL) (Morales et al., 2004; Gelbart and Kuroda, 2009), MOF was originally purified from a complex containing MSL (Bone and Kuroda, 1996). The MSL complex plays a significant role in balancing X-linked gene expression between male and female *drosophila*. The structure of MOF in mammals is significantly similar to the one in *drosophila*, which contains an acetyl-CoA binding site, a C2H2 zinc finger domain and a histone-binding chromatin region (Avvakumov and Cote, 2007). In mammals, MOF regulates the transcriptional activation through the formation of MSL and MOF-MSL1v1, which are evolutionarily conserved.

As an important histone acetyltransferase, MOF participates in several activities, such as gene transcription, chromatin stability, DNA damage repair, early embryonic development and tumorigenesis (Sharma et al., 2010; Li et al., 2012; Deng et al., 2013; Valerio et al., 2017; Yang et al., 2017). Few studies investigated the function of MOF in cancer. For instance, Valerio et al. (2017) reported that knocking out MOF in AML mice could relieve symptoms and prolong the life span, indicating that MOF may act as an oncogene in AML cells. Nevertheless, the biological effects and related mechanisms of MOF in thyroid cancer cells have not yet been elucidated.

With the increase morbidity and mortality of thyroid cancer, the most common disease in human endocrine system, research on pathogenesis and treatment of thyroid cancer is especially important. Thyroid cancer is a low-grade tumor (Vu-Phan and Koenig, 2014). The Human Epigenetics Association for Cancer Research launched the Human Epigenetic Program in 2003, which is aimed that understanding the epigenetic mechanisms of tumor (Jones and Martienssen, 2005). Gene expression profiling of differentiated thyroid cancer suggests that changes in DNA methylation, histone modifications, LncRNA and microRNA may contribute to tumorigenesis and tumor development (Asa and Ezzat, 2018). Currently, it is believed that HAT (Histone acetyltransferase) and HDAC (Histone deacetylase) are considered as novel anticancer targets (Noureen et al., 2010). Therefore, finding a target for thyroid cancer treatment through histone acetylation modification is promising.

In this study, we reported that MOF, which was upregulated in most thyroid cancer, activated the PI3K/AKT pathway through binding TNK2 promoter. MOF significantly promoted proliferation and inhibited apoptosis in thyroid cancer. Overall, these findings revealed a novel function of MOF in cancer. MOF may be a potential therapeutic target and diagnostic marker for thyroid cancer.

## Materials and Methods

### Patients and Tissue Samples

The retrospective research included 20 cases of thyroid cancer which were collected in Qilu Hospital from September, 2016 to June, 2017. The patients already gave written informed consent, which complied with the rules of the Ethics Committee of Shandong University. The thyroid cancer tissue chip was bought from Guge biotechnology company (Wuhan, China).

### Cell Culture and Transfection

N-thy-ori, BHP-10-3, IHH-4, TT2609 and 8505C cell lines were obtained from the American Type Culture Collection (ATCC, United States) and were, respectively, maintained in 1640/1640/DMEM/F12K/MEM with 10% fetal bovine serum (FBS, Gibco, United States). All cells were cultured at 37°C with CO_2_ in a humidified incubator. We knocked down MOF in BHP-10-3 and TT2609 cell lines by lentivirus infection. For stable infection, cells were infected with lentivirus for 24 h and then selected for 2 weeks in medium containing 2 μg/ml puromycin to acquire stable expression cells.

### Cell Proliferation Assay

The monoclonal cells and control cells were plated in a 96-well plate at 2 × 10^3^ cells/well once a day for 6 days. On the sixth day, these cells were treated with 10 μL of 5 mg/ml CCK8 (APExBIO, United States) per well for 4 h at 37°C with CO_2_ incubator. Viable cells were counted by reading the absorbance at 450 nm using a microplate reader.

### Plate Colony Formation Assay

The two monoclonal cell lines were plated in 10 cm dishes at 200 cells/well. After 10 days of culture, the cells were washed by PBS and stained by Giemsa. The colony number was counted under the light microscope, with one colony containing more than 50 cells. Each experiment was repeated in triplicate.

### EdU Staining Assay

The cells were incubated with EdU labeling solution (Abcam, United States) for 2 h at 37°C with CO_2_, then added with 2 mg/ml glycine to terminate the fixing. The staining was carried out according to the procedure on the kit, and finally the staining was observed with a fluorescence microscope.

### Cell Cycle Assay

The stable expression cells and control cells were collected and washed twice with PBS. Cells were added with 1 ml PI per 10^6^ cells protected from light for 30 min. Cell cycle was assessed by flow cytometer.

### Annexin V-FITC/PI Apoptosis Assay

The stable expression cells and control cells were collected and washed twice with PBS. Cells were diluted with 400 μL binding buffer. The cell suspension was then added with Annexin V-FITC and incubated at 4°C for 15 min protected from the light. Then the cell suspension was added with PI for 30 min.

### Western Blot Analysis

In order to detect the relative protein expression, BC200 lysis buffer was used for western blot. The protein samples were separated by SDS-PAGE and electro-transferred onto PVDF membrane, and then incubated with monoclonal anti-human MOF, H4K16ac, p21, p16, Cyclin D1, Cyclin D2, BAX, BCL2, p-BAD, AKT, p-AKT, GAPDH (Abcam, United States) at 4°C overnight. The membranes were probed with anti-mouse/rabbit IgG (Abcam, United States) at 1:5,000 dilution for 1 h, and then added with ECL reagent for examine.

### RNA Isolation and qPCR

Total RNA was extracted from cultured cells or fresh tissues with Trizol (TaKaRa, Japan) reagents following the kit protocol. And then, the total RNA was reverse transcribed into cDNA by using the RevertAid First Strand cDNA Synthesis Kit (Thermo, United States). qRT-PCR was performed to detect mRNA expression by using the Bio-Rad CFX96 real-time PCR detection system and SYBR Premix ExTaq Kit (TaKaRa, Japan). The primer sequences tested for qRT-PCR are shown in Supplementary Table S1.

### Immunohistochemistry Staining

To detect the expression level of MOF in thyroid cancer tissues, immunohistochemical staining of thyroid adjacent cancer tissue chips was performed. The tissue sections were deparaffinized in xylene and rehydrated in alcohol. Endogenous peroxidase activity in the tissue sections was blocked in 3% H_2_O_2_ for 15 min in the dark. The tissue was subjected to antigen retrieval using sodium citrate buffer by microwave, and naturally cooled to room temperature. 5% BSA was used to block the tissues at 37°C for 1 h. The tissues was incubated with primary antibody (anti-MOF) at 4°C overnight, and then was washed thrice by PBS. Secondary antibody was incubated with the tissues at 37°C for 1 h. After thrice washing with PBS, the tissues was stained by DAB and Hematoxylin. Images of the tissues were taken by using a light microscope (Olympus, Japan).

### Chromatin Immunoprecipitation Assay

ChIP assay was performed by referring to the ChIP kit (CST, United States) protocol. The DNA of cells were then immunoprecipitated with anti-MOF antibody. The purified DNA was analyzed by qRT-PCR. Sequences of all primer pairs used in the research are listed in Supplementary Table S2.

### Statistics Analysis

The experimental data were expressed as mean ± standard deviation (Mean SD), analyzed by GraphPad Prism5 software. The data comparison between groups was based on the principle of *t*-test, and *p* value < 0.05 was considered statistically significant standard.

## Results

### MOF Was Upregulated in Most Thyroid Cancer

**FIGURE 1:**
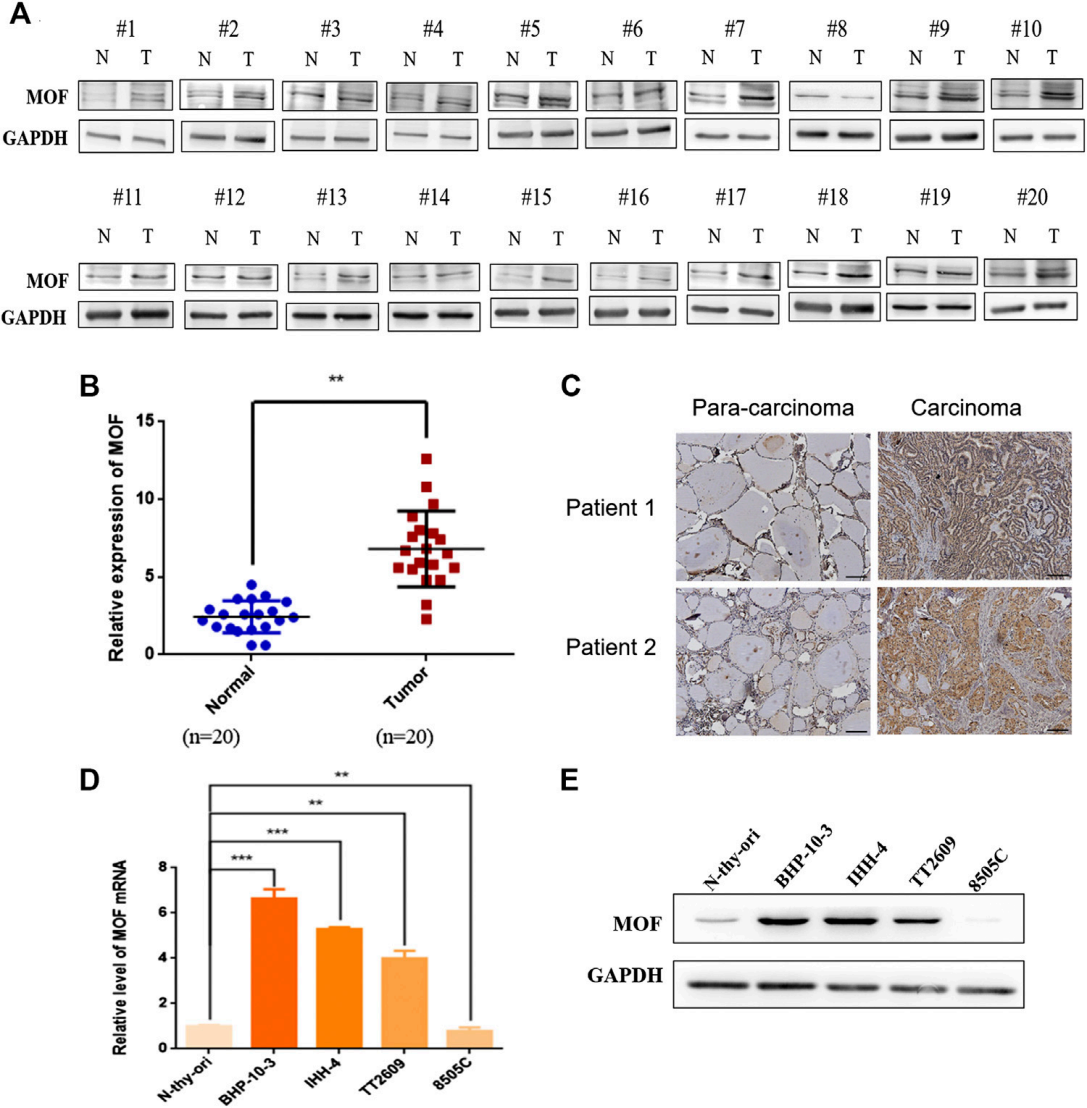
Expression analysis of MOF in thyroid cancer. **(A**,**B)** Western blot analysis of MOF in thyroid cancer tissue samples (n = 20) compared with normal fimbria (n = 20). **(C)** Representative images of immunohistochemical staining of MOF in tissue chip. **(D)** The MOF expression were measured by qRT-PCR in N-thy-ori, BHP-10-3, IHH-4, TT2609 and 8505C cell lines. **(E)** Western blot analysis of MOF in N-thy-ori, BHP-10-3, IHH-4, TT2609 and 8505C cell lines. **p < 0.01, ***p < 0.001.

To ascertain the expression of MOF in thyroid cancer, western blot was performed in 20 thyroid cancer tissue samples and matched corresponding normal tissue. Expression of MOF was significantly up regulated in thyroid cancer tissue (Figures 1A,B). We calculated the clinicopathologic features of 20 patients (Table 1). Immunohistochemical staining was performed and the results showed that cancer tissues with high MOF expression accounted for 96.6% (28 cases) compared with adjacent tissues, and cancer tissues with low MOF expression accounted for 3.4% (1 case) (Figure 1C). We analyzed the clinicopathologic parameters of 29 patients, and found something, which was consistent with the statistical results of these 20 patients (Table 2). To further demonstrate the expression of MOF in thyroid cancer, qRT-PCR and western blot were performed in N-thy-ori, BHP-10-3, IHH-4, TT2609, 8505C cell lines. Compared with the normal cell line N-thy-ori, MOF had a high expression in BHP-10-3, IHH-4, TT2609 cell lines (Figures 1D,E). The results demonstrated that MOF was upregulated in most thyroid cancer cell lines. Therefore, we suspected that MOF may play an important role in the development of thyroid cancer.

**TABLE 1:** Qilu Hospital 20 patients characteristics and association with MOF expression.

Variable	Category	No. of patients	%
MOF expression	High	18	90.0%
	Low	2	10.0%
Sex	Male	4	20.0%
	Female	16	80.0%
Age	<45	9	45.0%
	≥45	11	55.0%
Histology	Typical PTC	18	90.0
	Follicular subtype PTC	2	10.0

**TABLE 2:** Patient characteristics and association with MOF expression in pathology chip.

Variable	Category	No. of patients	%
MOF expression	High	28	96.6%
	Low	1	3.4%
Sex	Male	8	27.6%
	Female	21	72.4%
Age	<45	15	51.7%
	≥45	14	49.3%
Histology	Typical PTC	28	96.6%
	Follicular subtype PTC	1	3.4%

### Knockdown of MOF Inhibited Cell Proliferation

To investigate the functional role of MOF in thyroid cancer, we established two MOF knockdown cell lines by lentivirus infection and examined the effect of MOF on cell proliferation and apoptosis.

**FIGURE 2:**
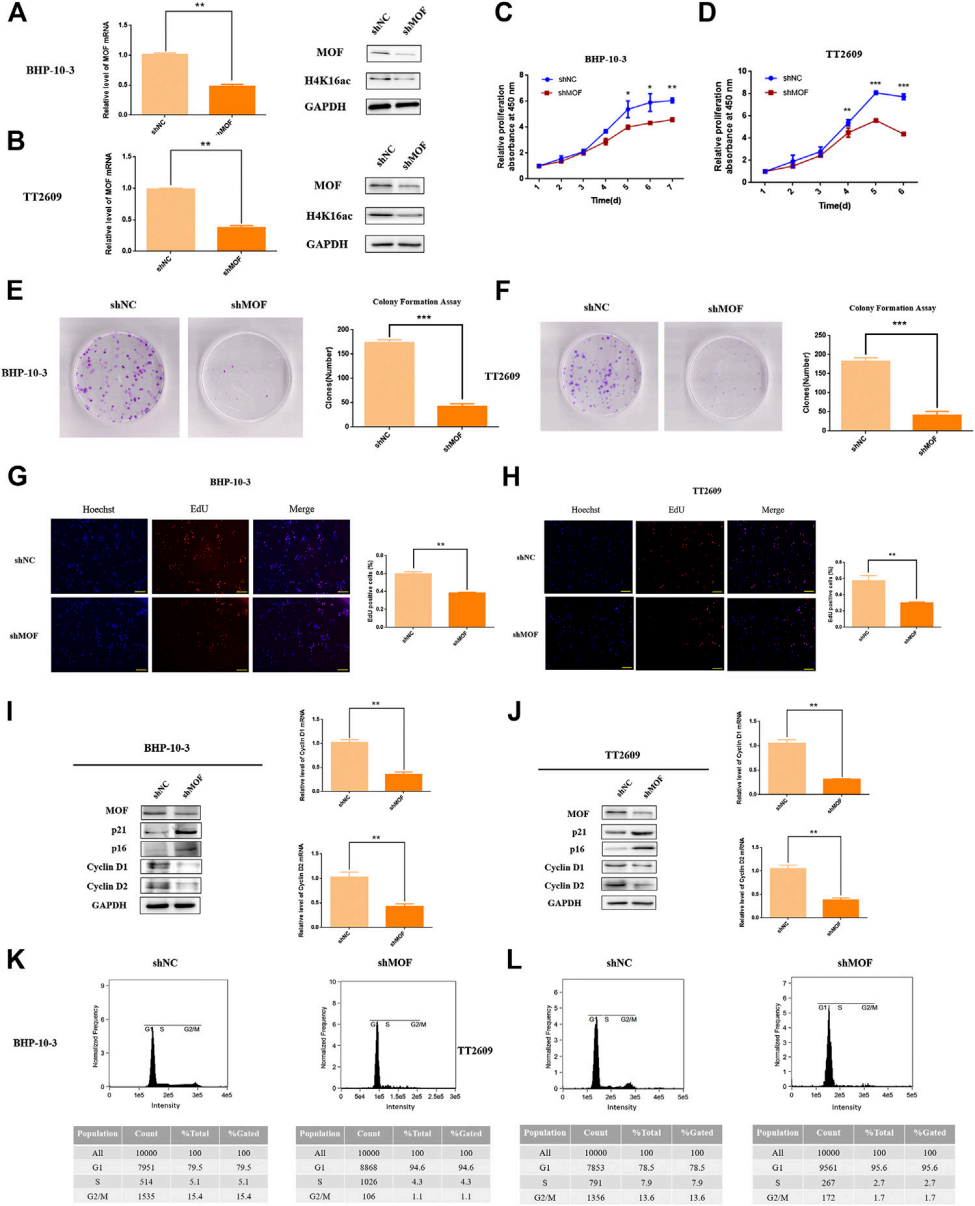
MOF inhibited the proliferation and colon formation ability of thyroid cancer cells. **(A**,**B)** Decreased MOF mRNA and protein expression in stable MOF-knockdown BHP-10-3 and TT2609 cells were detected and verified. **(C**,**D)** CCK8 assay of stable MOF-knockdown cell line showed decreased proliferation ability. **(E**,**F)** Colony formation assay of MOF-knockdown or control BHP-10-3 and TT2609 cells. **(G**,**H)** EdU staining assay of stable MOF-knockdown or control BHP-10-3 and TT2609 cells. **(I**,**J)** Cyclin D1, Cyclin D2, p21 and p16 mRNA and protein expression in stable MOF-knockdown BHP-10-3 and TT2609 cells were performed by Western blot and qRT-PCR. **(K**,**L)** Cell cycle was measured using PT staining assay by FCM. *p < 0.05, **p < 0.01, ***p < 0.001.

MOF was knocked down in BHP-10-3 and TT2609 cells and efficacy was verified (Figures 2A,B). It is found that cell proliferation ability was decreased after knocking down MOF by CCK8 assay (Figures 2C,D). Accordingly, EdU incorporation assay showed that knockdown of MOF reduced the DNA synthesis rate thus inhibits cell proliferation (Figures 2G,H). Furthermore, we detected p21, p16, Cyclin D1 and Cyclin D2 by western blot and qRT-PCR in stable and control cells. Cyclin D1 and Cyclin D2 expression were obviously decreased after knocking down MOF, while p21 and p16 were upregulated (Figures 2I,J). Through PI staining by FCM, the G1 phase was significantly blocked in two knockdown cell lines (Figures 2K,L). Besides, decreasing the expression of MOF also affected the colony formation ability (Figures 2E,F).

### Knockdown of MOF Promoted Apoptosis

**FIGURE 3:**
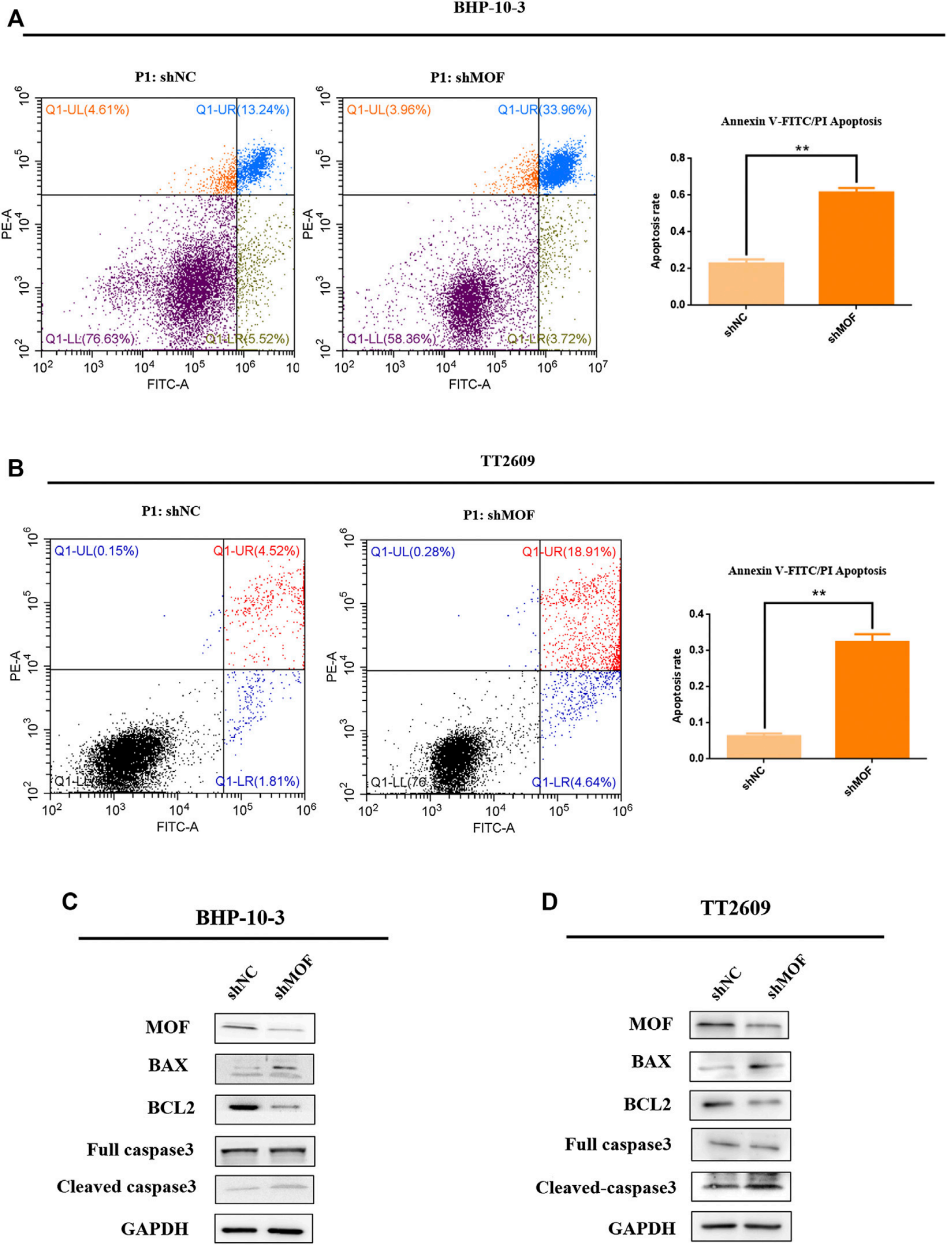
Knockdown of MOF promoted the apoptosis of thyroid cancer cells. **(A**,**B)** In BHP-10-3 and TT2609 cell lines, the effect of knockdown MOF were measured using PI/FITC staining assay. **(C**,**D)** Western blot assay was performed to detect the protein expression of apoptosis marks including caspase 3, cleaved-caspase 3, BAX, BCL2 in stable MOF-knockdown and control BHP-10-3 and TT2609 cells. MOF regulated TNK2 transcriptional expression in thyroid cancer cells.

Influence of MOF on apoptosis was detected in order to investigate its role on malignant characteristics of the thyroid tumor. Knockdown cell lines with corresponding control groups were stained with PI and FITC, which were analyzed by flow cytometer for apoptosis based on DNA content (Figures 3A,B). It is found that the apoptosis percentage was significantly increased after knocking down MOF comparing with the control group. What’s more, western blot analyze was performed to elevate the levels of apoptosis markers, including caspase 3, cleaved-caspase3, BAX and BCL2 (Figures 3C,D). Overall, knockdown of MOF promoted cell apoptosis and inhibited development of thyroid tumor.

**FIGURE 4:**
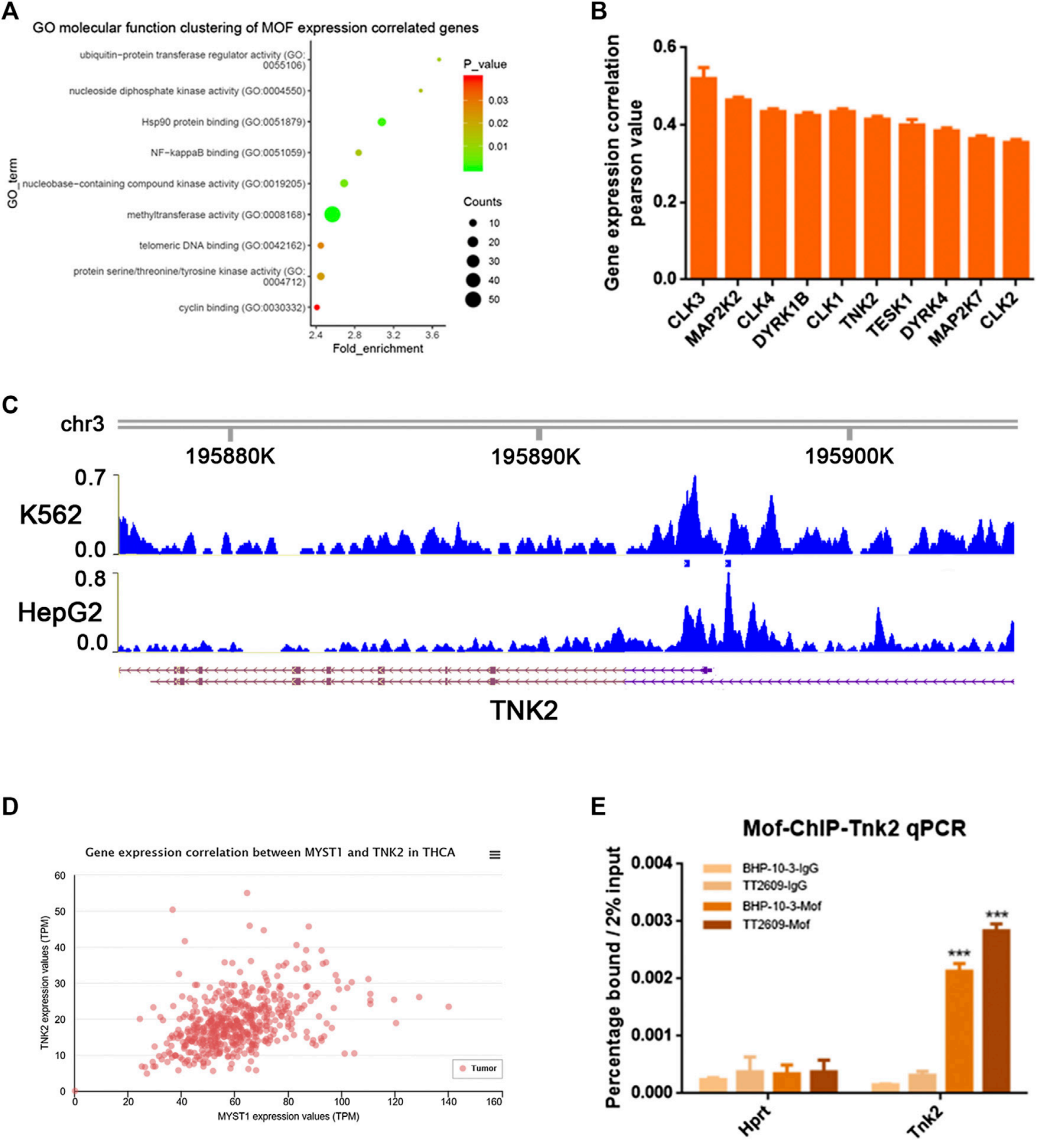
MOF bound to the phosphokinase TNK2 promoter. **(A)** Cluster of MOF expression correlated genes were enriched and classified according to GEO molecular functions. **(B)** Analyze the correlation between phosphokinase and MOF expression in TCGA database. **(C)** Screen promoters that might bind to MOF by Cistrome DB database. **(D)** Analyze the correlation between TNK2 and MOF expression in TCGA database. **(E)** ChIP analysis showed the occupancy of MOF on the TNK2 promoter *in vivo*. *p < 0.05, **p < 0.01, ***p < 0.001 Table 1. Qilu Hospital 20 patients characteristics and association with MOF expression.

MOF acted as an oncogene function in thyroid cancer. In GEO database, we selected the GO molecular function clustering of MOF expression correlated genes, and annotated the functions of these genes. It was clear that there are more than 20 genes which was related to protein serine/threonine/tyrosine kinase activity (Figure 4A). We screened some phosphokinases that are positively correlated with MOF expression in the cancer genome atlas (TCGA) database, as shown in Figure 4B. Through analyzing the previous MOF transcriptome and ChIP-Seq data in K562 and HepG2 cell lines in Cistrome DB database, several potential MOF targets phosphokinases were discovered, including TNK2, DYK1B and MAP2K2. Both of them had MOF binding peak in their promoter region (Figure 4C; Supplementary Figures S1C,D). We verified the correlation between TNK2, DYK1B, MAP2K2 and MOF expression in the TCGA database, and found that in thyroid cancer cells, they all had a strong positive correlation with MOF expression (Figure 4D; Supplementary Figures S1A,B). With the assumption that MOF could regulate several downstream targets by transcription activation, we supposed that MOF could bind the promoter region of TNK2, DYK1B, MAP2K2. We conducted chromatin immunoprecipitation (ChIP) assay in BHP-10-3 and TT2609 cells. After cross linking and chromatin fractionations the DNA-protein complex was immunoprecipitated with MOF and IgG antibody. We observed occupancy of MOF at the TNK2 promoter, whereas little signal was amplified from the IgG immunoprecipitated negative control by qRT-PCR (Figure 4E). Compared with the negative control, it had little change between DYK1B, MAP2K2 and MOF combination (Supplementary Figures S1E,F). In summary, MOF might bind to the promoter of TNK2 and mediate its transcriptional activation.

### MOF Could Mediate the Activation of PI3K/AKT Signaling Pathway Caused by Phosphorylation of AKT by TNK2

**FIGURE 5:**
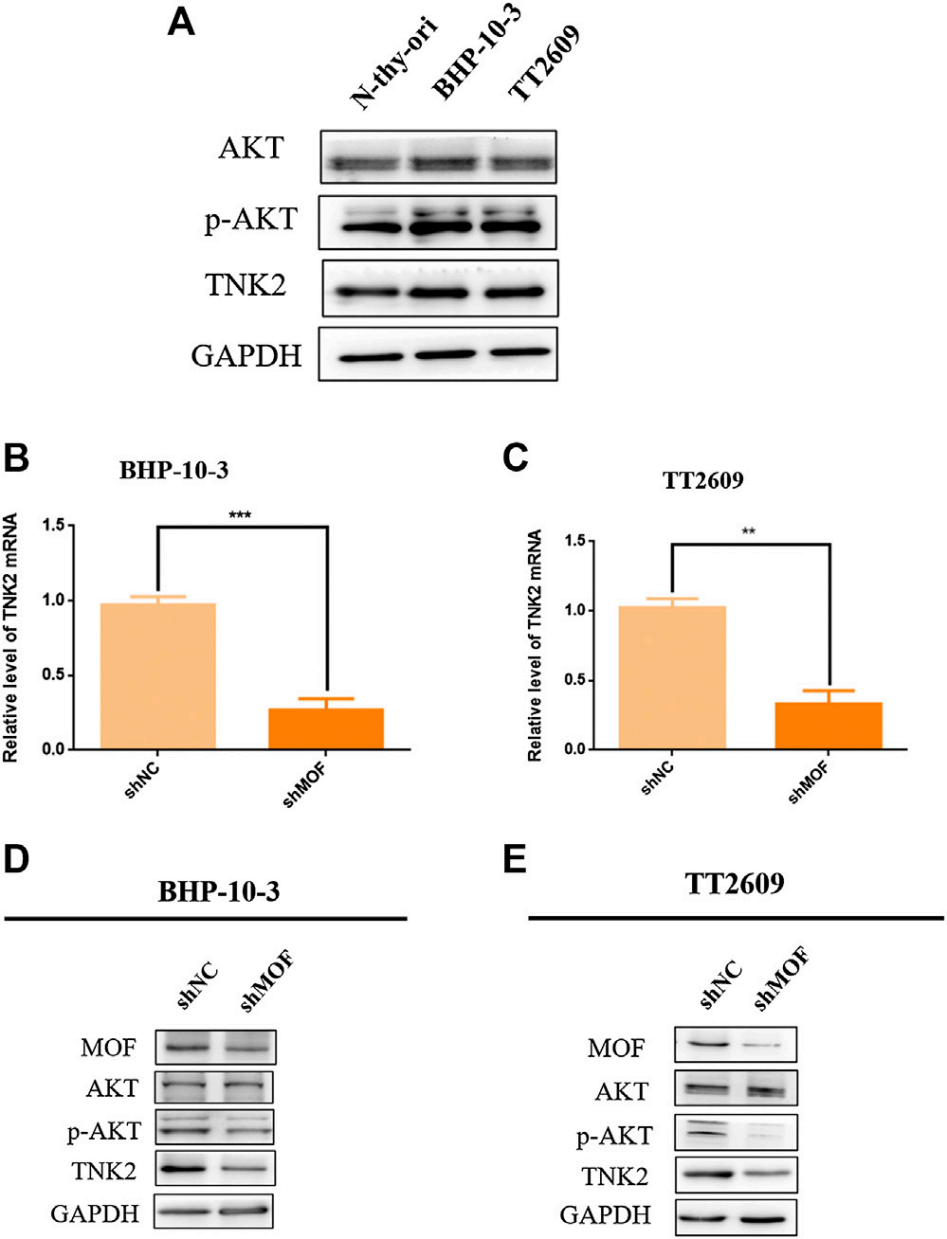
MOF bound to TNK2 promoter element to mediate PI3K/AKT pathway. **(A)** Western blot assay was performed to detect the protein expression of PI3K/AKT pathway in N-thy-ori, BHP-10-3, TT2609 cell lines. **(B**,**C)** TNK2 mRNA expression in stable MOF-knockdown BHP-10-3 and TT2609 cells were detected by qRT-PCR. **(D**,**E)** Western blot analysis of TNK2, AKT and p-AKT in stable MOF-knockdown BHP-10-3 and TT2609 cells. *p < 0.05, **p < 0.01, ***p < 0.001. PI3K/AKT pathway was inhibited with MG149 treatment in thyroid cancer cells.

TNK2 is a specific AKT phosphokinase (Mahajan and Mahajan, 2015). Compared with thyroid cell lines N-thy, TNK2 and p-AKT was upregulated in thyroid cancer cell lines BHP-10-3 and TT2609 (Figure 5A). TNK2 mRNA expression was accurately quantified with real-time PCR assay in MOF-knockdown or control cells of BHP-10-3 and TT2609 (Figures 5B,C), which showed considerable down regulation of TNK2 expression after MOF knockdown. What’s more, we detected the expression of AKT, p-AKT and TNK2 by western blot in stable MOF-knockdown cell lines. As was shown in Figures 4D,E, knockdown of MOF could inhibit the PI3K/AKT pathway by blocking the protein expression of TNK2 and p-AKT, which was related with the cell proliferation and apoptosis. The conclusion was consistent with our previous results that the proliferative capacity of the cells was reduced and the level of apoptosis was increased after knocking down MOF in BHP-10-3 and TT2609.

**FIGURE 6:**
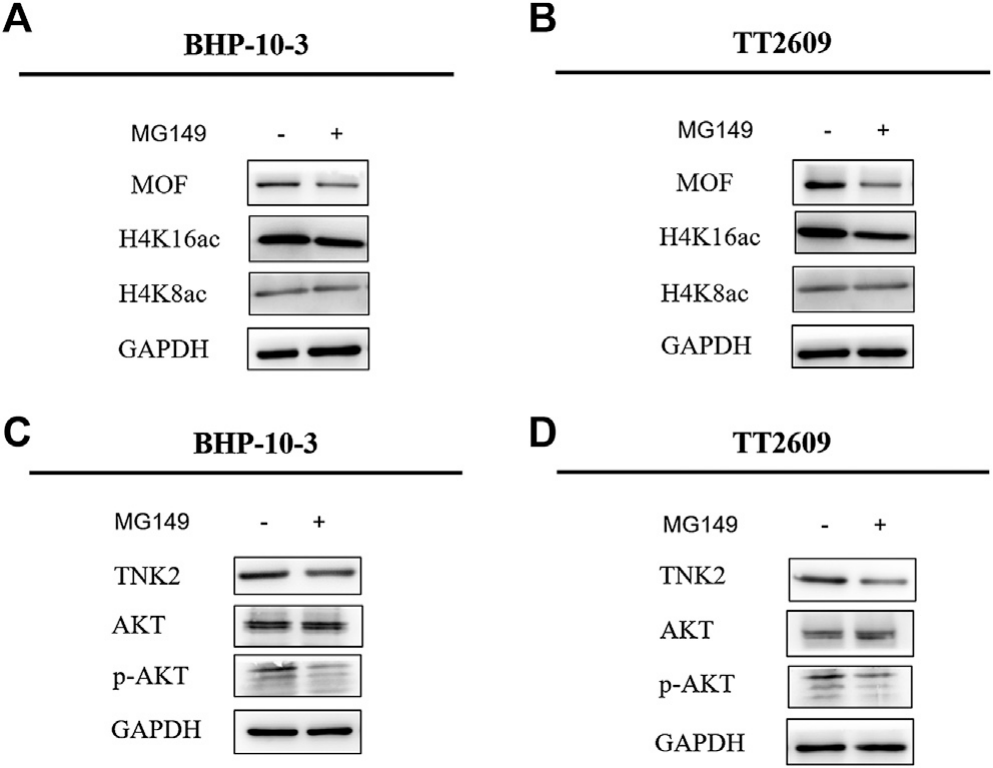
MG149 treatment in BHP-10-3 and TT2609 cell lines reduced the expression of TNK2 and p-AKT. **(A**,**B)** Western Blot detected the expression of MOF, H4K16ac, and H4K8ac levels after treatment of BHP-10-3 and TT2609 cell lines with 33um MG149. **(C**,**D)** Western blot showed the PI3K/AKT pathway could be reduced by MG149 treatment in BHP-10-3 and TT2609 cell lines.

MG149 was a histone acetyltransferase inhibitor, which could act on several histone acetyltransferases like MOF, Tip60 etc. Optimal MG149 concentration for MOF was determined as 33 μm. The expression of H4K16ac was obviously inhibited and H4K8ac was constant through Western Blot assay in BHP-10-3 and TT2609 cell lines (Figures 6A,B). What’s more, the phosphorylation level of AKT was reduced in the two treated cell lines through western blot assay, which was corresponding to the result after knocking down MOF (Figures 6C,D). It was demonstrated that inhibition of MOF enzyme activity could also decrease the expression of TNK2 and affect the PI3K/AKT signaling pathway represented by the reduced phosphorylation level of AKT. Therefore, our findings revealed the feasibility that MOF could be considered as a target for thyroid cancer treatment.

## Discussion and Conclusion

The present study identified a novel function of MOF in progression of thyroid cancer. MOF is member of the histone acetyltransferase MYST family, which not only specifically acetylates histone H4K16 but also non-specifically acetylates p53, NRF2 and other genes. The acetylation of p53 by MOF leads to the up-regulation of mRNA levels of the apoptotic genes BAX and PUMA (Li et al., 2009). And acetylation of NRF2 leads to nuclear arrest of NRF2, which in turn affects the transcription level of NRF2 target gene (Chen et al., 2014). The biological activity of MOF determines its diverse biological functions, and MOF plays an important role in many life span such as self-renewal of embryonic stem cells, gene transcription, maintenance of chromosomal stability, immune inflammatory response, and tumorigenesis. The expression of MOF is upregulated in non-small lung cancer. The mechanism is that MOF blocks its nucleation by acetylation of NRF2, affecting the downstream signaling pathway, which is different from that of MOF in thyroid cancer.

In this study, we first analyzed the expression of MOF in thyroid cancer pathology and cell lines, showing that MOF is up-regulated in most thyroid cancers including papillary thyroid cancer and follicular thyroid cancer. So, we uncovered the precise function and mechanism of MOF in thyroid cancer. Results showed that MOF significantly promoted proliferation and inhibited apoptosis. We knocked down MOF in the papillary thyroid cancer cell line BHP-10-3 and the follicular thyroid cancer cell line TT2609 by lentivirus infection, and two monoclonal MOF-knockdown cell lines were screened by puromycin subsequently. After knocking down MOF, the level of acetylation of H4K16 was significantly decreased, which showed its effect on epigenetic modifications. According to CCK8, colony formation and EdU staining we could draw a conclusion that the proliferation ability of knockdown MOF cells was inhibited. Expression of cell proliferation inhibitory proteins p21 and p16 were up-regulated and cyclin D1, cyclin D2 expression were down-regulated. Mammalian cells encodes three D cyclins (D1, D2, D3) that coordinately function as allosteric regulators of cyclin-dependent kinase 4 (CDK4) and CDK6 to regulate cell cycle transition from G1 to S phase (Goel et al., 2017). Cyclin D1 is more frequently abnormal than cyclin D2 or D3 in human tumors, so it has been more widely characterized (Qie and Diehl, 2016). According to the down regulation expression of cyclin D1 and D2 in knockdown MOF cells, We detected the cell cycle by PI staining and flow cytometry. Based on BN Sheikh’s research (Sheikh et al., 2016), we speculated that the reason why G1 phase blocked was that MOF directly bound to genes required for cell cycle progression and maintained their transcription. However, further research is needed on the specific mechanism which cyclin D1 regulates the change of thyroid cancer cell cycle.

Apoptosis is a process of programmed cell death, which is regulated through the balance between proapoptotic and antiapoptotic proteins like BCL2 family (Ghobrial et al., 2005). The family has many members including MCL-1, NR-B, BAX, BAD, BIM, BCL-XL, etc. Most members of BCL2 family have two structural homology regions, which play an important role in mediating the dimerization process (Radha and Raghavan, 2017). BAX is an important proapoptotic proteins, and BCL2 is a crucial proapoptotic protein. The ratio of BCL2/BAX can reflect apoptotic activity (Qiao and Wong, 2009). BCL2 can inhibit this process by suppressing the translocation of BAX, decreased the activity of the caspases (Huang et al., 2016). Caspase 3 is type of proteinase. When caspase 3 is activated, it can require proteolytic processing its inactive zymogen into activated p17 and p12 fragments, to play a central role in the execution-phase of cell apoptosis (Nagata, 2018). Flow cytometry showed that the apoptosis level of MOF-knockdown stable cells was significantly increased. What’s more, the apoptosis-promoting protein BCL2 was up-regulated, and BAX which could inhibit apoptosis was down-regulated. Total expression of caspase 3 changed inapparently, but the p17-caspase 3 was obviously up-regulated. However, studies have shown that MOF also plays an important role in autophagy (Füllgrabe et al., 2017), so further research is needed on the effects of excluding autophagy in apoptotic cells.

In order to explore the mechanism of MOF as an oncogene in thyroid cancer, we searched the TCGA database for genes that were positively related to MOF, and annotated the molecular functions of these genes based on the GEO database. We found protein serine/threonine/tyrosine kinase activity was related to MOF. According to that MOF is a well-known transcriptional coactivator for regulating the expression of target genes (Yang et al., 2018), we attempted to confirm that TNK2, DYK1B and MAP2K2 may be a target of MOF at the transcriptional regulation in thyroid cancer. Therefore, ChIP research was performed in BHP-10-3 and TT2609 cell lines, demonstrating that MOF binds to the promoter region of TNK2, thereby directly regulating the transcriptional expression of TNK2. Based on the above results, the downstream signaling pathway PI3K/AKT was further explored. The protein expression of TNK2 and p-AKT were significantly up-regulated in BHP-10-3 and TT2609 cell line compared to normal thyroid cell lines N-thy-ori. Given that TNK2 could directly phosphorylate AKT to activate the PI3K/AKT pathway (Datta et al., 1997), we detected the expression of TNK2 and p-AKT in knock-down MOF cell lines. The TNK2 expression was significantly down-regulated, resulting in a decrease in the level of p-AKT with MOF. Treatment of BHP-10-3 and TT2609 with histone acetyltransferase inhibitor MG149 showed similar results with that knocking down MOF, further illustrating the possibility of MOF as a target in treatment of thyroid cancer. To sum up, our results revealed that MOF could bind to TNK2 promoter which activated the PI3K/AKT pathway, highlighting the extensive significance of MOF on tumor development and treatment.

This study revealed that MOF played a role in thyroid cancer as an oncogene and promoted the development of tumor. However, knocking down MOF in thyroid cancer cell lines only unilaterally verified the changes in proliferation and apoptosis of thyroid cancer cells. Whether overexpression of MOF in thyroid cancer cell lines can accelerate the development of thyroid cancer requires further verification. The research was studied *in vitro*, and further research *in vivo* is needed, such as animal experiments and even clinical trials. It has been reported that VEGFR inhibitors have become the most commonly molecular targeted therapeutic drugs for clinical treatment of differentiated thyroid cancer, which can effectively reduce tumor cell proliferation and angiogenesis (Schlumberger et al., 2015), but are prone to certain side effects and resistance (Cabanillas et al., 2017). Considering our result, the inhibitor of MOF combining with VEGFR inhibitor in treatment of thyroid cancer may reduce the cell proliferation ability and accelerate the apoptosis of cancer cells, leading to less side effect, which also needs further study.

No research provided complete information on roles of MOF in thyroid tumorigenesis and tumor progression. In summary, our study found that histone acetyltransferase MOF is up-regulated in most thyroid cancers, revealing that MOF plays an important role in the proliferation and apoptosis of thyroid cancer cells. This effect is based on the direct transcriptional activation of TNK2 by MOF and change of downstream PI3K/AKT signaling pathway. Increased MOF expression may be a key event in thyroid cancer progression, such that MOF may be regarded as a potential prognostic marker for thyroid cancer.

## Data Availability Statement

The datasets presented in this study can be found in online repositories. The names of the repository/repositories and accession number(s) can be found in the article/Supplementary Material.

## Ethics Statement

The studies involving human participants were reviewed and approved by School of Life Sciences ehtics committee, Shandong University. The patients/participants provided their written informed consent to participate in this study.

## Author Contributions

XL designed the experiments. DL performed the experiments. YY and BC helped perform experiments. XG and SG helped analyze datas. MW and MD offered kind advice.

## Funding

This study is supported by the National Key R&D Program of China (2016YFE0129200), the National Natural Science Foundation of China (No. 31571321 No. 81800727 and No. 81873632).

## Conflict of Interest

The authors declare that the research was conducted in the absence of any commercial or financial relationships that could be construed as a potential conflict of interest.
