# Supplementary figures and images for: MOF Regulates TNK2 Transcription Expression to Promote Cell Proliferation in Thyroid Cancer
Source: Front Pharmacol. 2020 Dec 8;11:607605. doi: 10.3389/fphar.2020.607605 (PMC7845732; doi:10.3389/fphar.2020.607605)

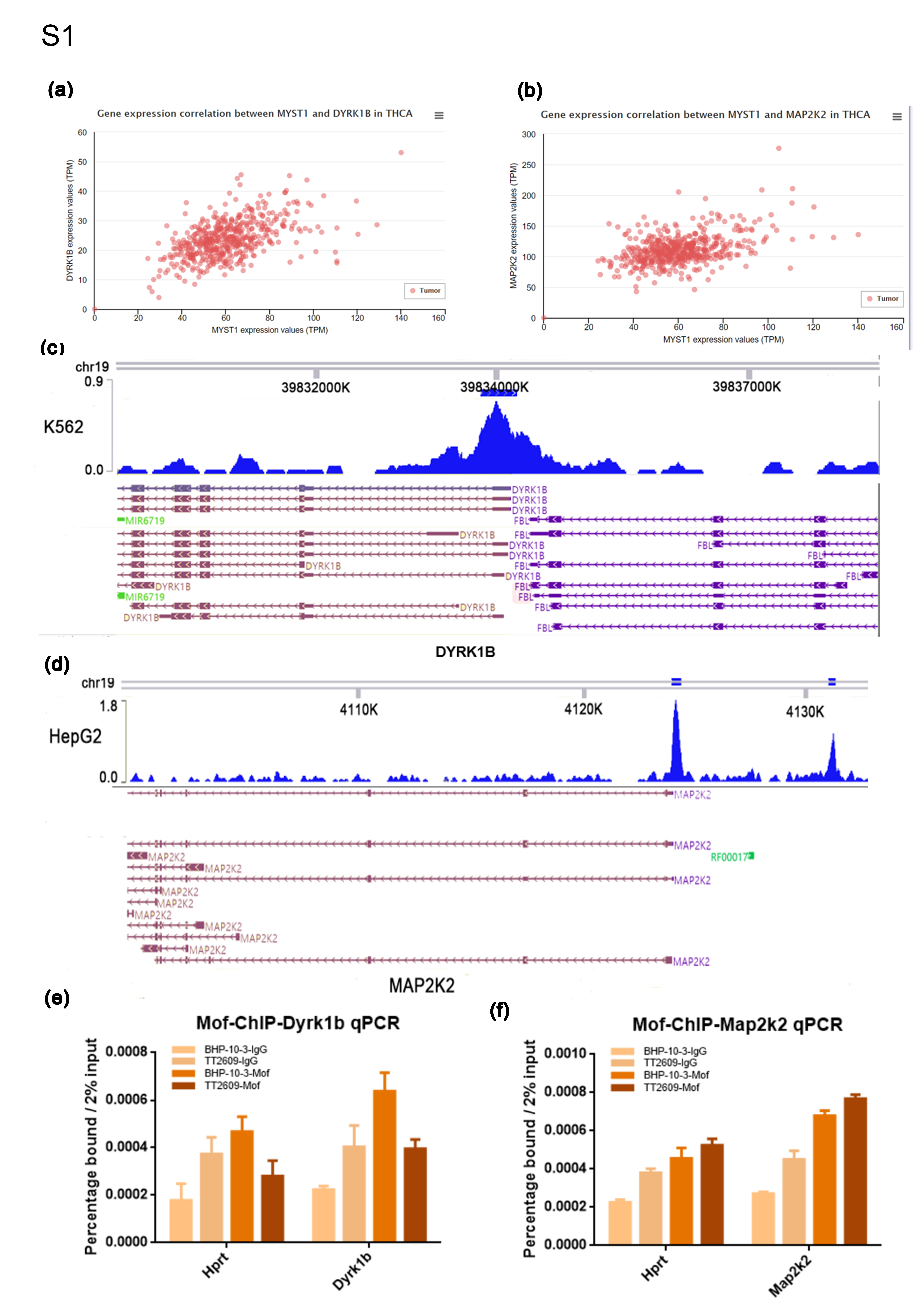

Supplement: Supplementary file 1 [file image1.jpeg]
